# Enhanced photovoltaics inspired by the fovea centralis

**DOI:** 10.1038/srep08570

**Published:** 2015-02-24

**Authors:** Gil Shalev, Sebastian W. Schmitt, Heidemarie Embrechts, Gerald Brönstrup, Silke Christiansen

**Affiliations:** 1 Max Planck Institute for the Science of Light, Günther-Scharowsky-Straße 1, 91058 Erlangen, Germany; 2 Helmholtz-Zentrum Berlin für Materialien und Energie, Kekulestrasse 5, 12489 Berlin, Germany

## Abstract

The fovea centralis is a closely-packed vertical array of inverted-cone photoreceptor cells located in the retina that is responsible for high acuity binocular vision. The cones are operational in well-lit environments and are responsible for trapping the impinging illumination. We present the vertical light-funnel silicon array as a light-trapping technique for photovoltaic applications that is bio-inspired by the properties of the fovea centralis. We use opto-electronic simulations to evaluate the performance of light-funnel solar cell arrays. Light-funnel arrays present ~65% absorption enhancement compared to a silicon film of identical thickness and exhibit power conversion efficiencies that are 60% higher than those of optimized nanowire arrays of the same thickness although nanowire arrays consist of more than 2.3 times the amount of silicon. We demonstrate the superior absorption of the light-funnel arrays as compared with recent advancements in the field. Fabrication of silicon light-funnel arrays using low-cost processing techniques is demonstrated.

In order for silicon-based solar energy to become a primary energy source it is essential to render it cost effective as compared with current carbon-based technologies^1^. The high costs stem from both the high grade silicon needed in order to provide the required electrical performance and sufficient bulk material to maximize light absorption to a level acceptable for sufficiently high efficiencies (~20% are required to compete with existing bulk and thin film technologies)^2^. For cost efficiency in novel crystalline silicon thin film technologies it is therefore mandatory to at least reduce the amount of the silicon used while maintaining power conversion efficiencies (PCE) of current wafer based technologies. Recently, crystalline silicon thin film solar cell technologies reached PCE of 11.5% and promising open circuit voltages (*V_oc_*) of 650 mV. However the cell design still suffers from insufficient trapping of visible and near infrared light^3^.

Nanostructuring the surface of a solar cells allows for a more efficient harvesting of solar radiation and also the collection of infrared radiation well below the absorption edge^4^. Silicon photovoltaics based on arrays of vertically-aligned cylindrical silicon nanowires (NWs) of several hundred nanometers in diameter with either radial or axial junction were demonstrated as potential candidates to address this challenge. These arrays exhibit appreciated absorption due to enhanced light trapping and support efficient carrier collection over short distances, and hence permit reduced thickness and the utilization of electrically low grade silicon (= low cost) with short minority carrier diffusion lengths (or lifetimes)^5^,^6^,^7^,^8^. Various arrays of silicon NWs were produced and demonstrated to potentially provide the required light absorption enhancement in thin layers, support tolerating short minority diffusion lengths, and are produced by means of low cost fabrication techniques^9^,^10^,^11^,^12^.

Another promising family of structures that are currently under examination for photovoltaics are either periodic or randomized vertical cone arrays. Cone arrays are reminiscent of the anti-reflective surfaces found on the transparent wings of hawkmoths and on corneas of moth and butterfly eyes, for example^13^,^14^. These surfaces consist of hexagonal arrays with periodicity of approximately 240 nm that are composed of subwavelength structures with rounded tips. Effectively, the subwavelength structures introduce a gradual refractive index profile and form a favorable optical impedance matching between the organic tissues and the surrounding air. Huang et al demonstrated silicon nanotip arrays mimicking the ‘moth eye’ array and reported sub 1% reflection with a tip height of 16 μm^15^. Recently, Jeong et al reported a record thin silicon solar cell efficiency of 13.7% attributed to enhanced absorption due to surface nanocone arrays, where an absorption of >95% for the wavelength range of 400–800 nm was shown for a 10 μm thick substrate^16^. Spinelli et al demonstrated a similar concept with arrays composed of nanocylinders residing on a silicon substrate for which the high levels of absorption are attributed to forward scattering by the cylinders into the substrate^17^.

In this paper, we introduce the light-funnel (LF) array which is a new light trapping scheme for photovoltaics that mimics the structure and arrangement of the cone photoreceptors in the fovea centralis region of the retina. The human retina, for example, has approximately 6 million cone photoreceptors and 120 million rod photoreceptors^18^. Their optical properties are determined by their size, morphological structure, as well as the media constituting and surrounding them^19^,^20^. Various papers described the wave guiding properties of these photoreceptors specifically with respect to mode formation^21^,^22^,^23^. Currently, open questions include micro anatomical and molecular differences between rods and cones^23^. Cones are operational in relatively bright light and are responsible for high acuity binocular vision, whereas rods photoreceptors trade acuity with sensitivity and are triggered in dim conditions. In that sense the bright light conditions required for the functionality of cones is reminiscent of the bright-light conditions required for the functionality of a solar cell while rods act more as sensitive light detectors. The fovea centralis is a distinct pit in the central retina containing only cones in a densely-packed mosaic that is recognized as the region responsible for high acuity binocular vision^24^. Moreover, the packing density of the foveal cones accurately correlates with the human visual acuity^18^,^25^. The geometrical difference between the retinal cones and the ‘moth eye’ cones is acute: the retinal cones are inverted with the large base facing the incoming illumination and can reach several microns in diameter and dozens of microns in length^19^,^26^ in contrast with the much smaller upright ‘moth eye’ cones.

We use three-dimensional (3D) Technology Computer Aided Design (TCAD) optical and electrical simulations to model periodic square-tiled arrays composed of LFs in the shape of inverted frustum cones. We demonstrate their superior absorption relative to both continuous thin films and NW arrays of identical thickness across a wide range of angles of incidence. Also, we present extensive electrical simulations showing that the overall PCE of the LF solar cell is 60% higher than the PCE of an optimized NW-based solar cell of the same thickness although the NW solar cell consists of more than 2.3 the amount of silicon. Furthermore, we demonstrate the superiority of the LF array against recent advancements in the field. Finally, fabrication of silicon LF arrays using low-cost fabrication techniques is demonstrated showing the technological feasibility of the proposed concept.

## Results and Discussion

### 3D finite-difference time-domain (FDTD) optical simulations

**Figure 1:**
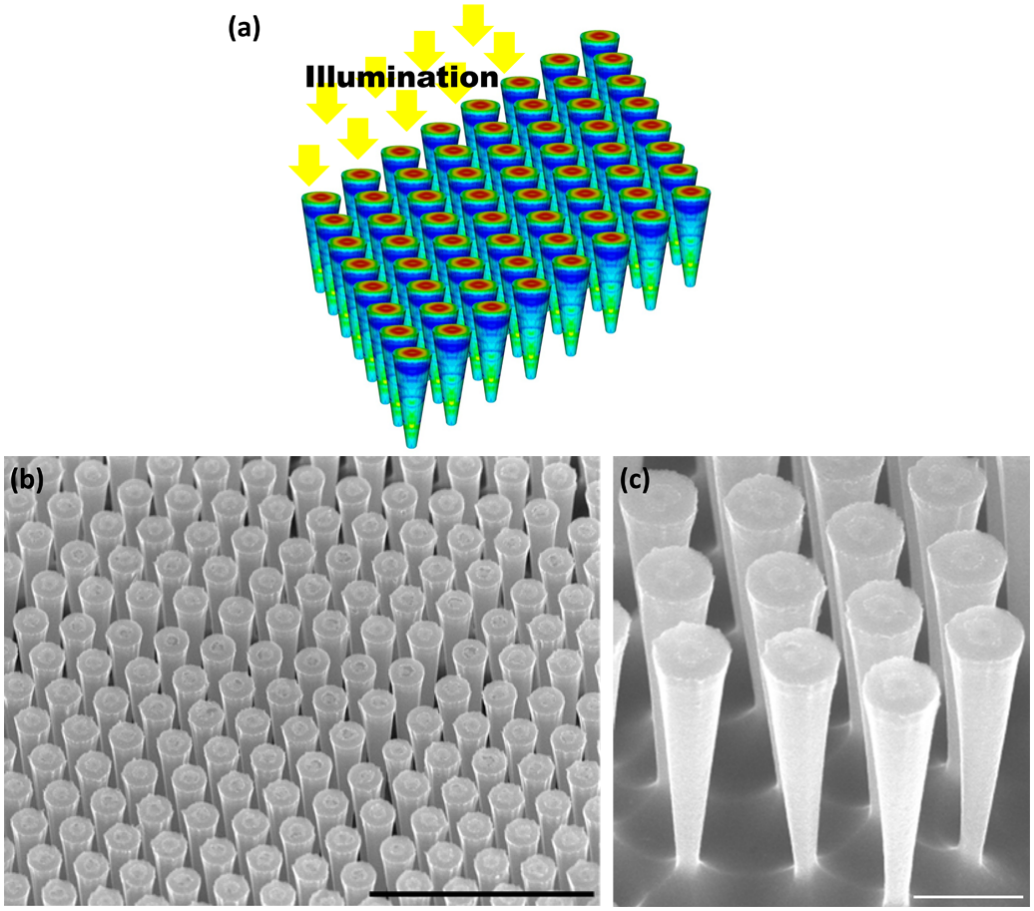
The light-funnel array. (a), 3D representation of a free-standing infinite square-tiled LF array under normal incidence where the color coded individual LFs represent the absorbed photon density. (b), A scanning electron microscope (SEM) image of a silicon LF array fabricated using low-cost fabrication techniques combining nano-sphere lithography and Langmuir-Blodgett deposition of 1 μm polystyrene spheres. Scale bar is 5 μm. (c), A patterned silicon wafer was cracked in order to image LFs from the side in order the better present their inverted frustum cone structure. Scale bar is 1 μm. See methods section for LF array fabrication.

**Figure 2:**
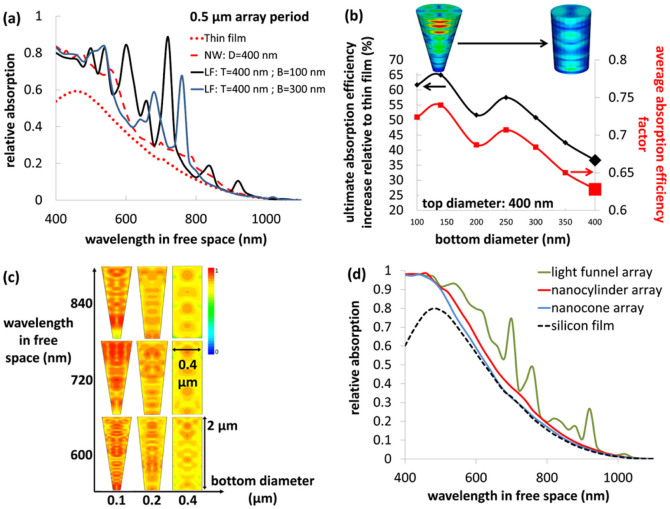
Optical absorption of a light-funnel array. (a), Relative absorption of LF arrays, a NW array and a continuous silicon thin film. The height is 2 μm. Note the absorption enhancement of the LF arrays. **Legend**: **T**: top diameter, **B**: bottom diameter. (b), Increase in ultimate absorption efficiency relative to a continuous silicon film and the absorption efficiency factor of a single LF (*Q_abs_*) in the array as a function of LF bottom diameter for a constant top diameter of 400 nm. Both the LF array absorption and the absorption efficiency factor of a single LF increase with decrease in bottom diameter despite the decrease in filling ratio. The period of the arrays is 500 nm and the heights are 2 μm. The large markers at bottom diameter = 400 nm reflect the values of a NW with the corresponding diameter. (c), The absorbed photon density of NWs and LFs at wavelengths 600 nm, 720 nm, 840 nm and for LFs bottom diameter of 100 nm, 200 nm, 400 nm. Note the increase in the absorbed photon density once deforming the 400 nm NW into a LF. (d), LF array: square-tiled array with a 520 nm period, *D_b_* = 100 nm, *D_t_* = 400 nm, 2 μ height and 50 nm Si_3_N_4_ anti-reflection coating. The geometries of the nanocone and the nanocylinder arrays are taken from the respective publications. Nanocone array: square-tiled array with a period of 500 nm, nanocone bottom diameter of 450 nm, nanocone height of 400 nm and 80 nm coating of SiO_2_. Nanocylinder array: square-tiled array with period of 450 nm, nanocylinder width of 250 nm, nanocylinder height of 150 nm, and 50 nm Si_3_N_4_ anti-reflection coating. Thin silicon film: 2 μm thickness with 80 nm standard anti-reflection coating. The thicknesses of the underlying substrates are selected to ensure that all three technologies share the same amount of silicon. Hence the thicknesses of the substrates are 1.12, 1.56 and 2 μm for the LF array, nanocone array, and the nanocylinder array, respectively.

Figure 1a presents an illustration of a 3D vertical LF square-tiled array where the color coded individual LFs reflect the absorbed photon density. Figures 1b,c show scanning electron microscopy images of LF arrays that were fabricated using low-cost fabrication techniques^27^ (see methods and Supplementary 1). Figure 2a presents 3D finite-difference time-domain (FDTD) optical simulations of the relative absorption spectra under normal incidence (Fig. 1) of two representative LF arrays with a period of 500 nm where the top diameter (*D_t_*) is held constant at 400 nm and the bottom diameter (*D_b_*) is fixed at 100 nm and 300 nm. The 500 nm period was selected as it couples best to the solar spectrum that peaks around wavelength of ~500 nm^10^ and therefore in the current work the considered array periods of both LF arrays and NW arrays are always set to 500 nm. For reference, the spectra of silicon thin film and of a NW array with NW diameter (*D*) of 400 nm, are shown as well. The juxtaposition of the LF array with a NW array not only serves for benchmarking but also allows a more acute understanding of the governing physics of the LF array. Unless otherwise specified the height (*h*) of all arrays and thin films is set to 2 μm. Thin film Fabry-Perot oscillations are not evident as the boundary condition at the bottom edge of the LF and the NW arrays and the thin film is set to perfectly matching layers (see methods). Note, that LF arrays exhibit the highest absorption. Furthermore, the LF arrays exhibit a broadband absorption similar to the NW array^12^,^27^,^28^,^29^,^30^ only superimposed with additional strong resonances at specific wavelengths. The ultimate absorption efficiency is the averaged and weighted (Air Mass 1.5) relative absorption, and it is assumed that each above bandgap photon generates an electron-hole pair that is collected at the electrodes. Figure 2b presents the ultimate absorption efficiency increase relative to a continuous silicon film for a LF array with *D_t_* of 400 nm vs. *D_b_*. The minimum considered *D_b_* is 100 nm, and the maximum considered *D_b_* is 400 nm which reflects the convergence of the LF into a vertical NW. Note that LF arrays exhibit an absorption enhancement of ~65% (for *D_t_* = 400 nm and *D_b_* = 140 nm) relative to the continuous film in comparison with NW arrays that present an absorption enhancement of 36.6%. Furthermore, the absorption of the LF array is superior despite the smaller filling ratio (defined as the volume of the LF divided by the volume of a unit cell) where 22% is calculated for the LF array with *D_t_* = 400 nm and *D_b_* = 100 nm and 50% for the NW array. The absorption enhancement of the LF array increases with the increase in cone angle (*α*) from 0° to 8°, which entails a decrease in filling ratio.

The relative absorption is a figure of merit describing the absorption performance of an array, while the absorption efficiency factor (*Q_abs_*) is a figure of merit describing the ability of a single particle (e.g. LF or NW) to concentrate or to couple light into itself. *Q_abs_* is the absorption cross-section (*C_abs_* - the ratio between the total absorbed photons and the incident photon flux) normalized by the LF top area^31^ (for normal incidence). Here *Q_abs_* is given by: *Q_abs_* = *C_abs_*/(π/4 *D_t_*^2^). The relative absorption and the *Q_abs_* of a LF or a NW nested inside an array relate to one another by the filling ratio. Figure 2b presents also the average of *Q_abs_* (averaged over the wavelength range of 400 nm–1100 nm) under normal incidence of a single LF in the array as a function of *D_b_*. Figure 2b shows that *Q_abs_* is directly correlated with the ultimate absorption efficiency and increases with a decrease in *D_b_*. Note that *Q_abs_* increase is due to increase in *C_abs_* as the area of the LF top base (i.e. π/4 *D_t_*^2^) that is used to scale *C_abs_* is not changed for all data points in Fig. 2b. The importance of this behavior becomes evident when considering the relocation of an isolated NW into a NW array; a single isolated subwavelength NW presents a relatively high *Q_abs_*^32^,^33^,^34^,^35^,^36^,^37^, however *Q_abs_* decreases substantially once the isolated NW is introduced into an array as adjacent NWs are now optically-coupled (Supplementary S2). This loss in *Q_abs_* of a single array-nested NW can be compensated by transforming the NW into a LF, decreasing the bottom edge of it and hence increasing *Q_abs_* and the overall array absorption as demonstrated in Fig. 2b where the *Q_abs_* of the 400 nm NW is increased from 0.63 to 0.72 for a light-funnel with *D_b_* = 100. Again, an increase in *Q_abs_* is not due to scaling of *C_abs_* but is due to increase in *C_abs_* itself which concludes an increase in the absorption of a single LF. Still, this ~15% increase of *Q_abs_* is not sufficient to account for the ~50% absorption enhancement of the light-funnel array with *D_b_* = 100 nm as compared with the 400 nm NW array. Hence, the absorption enhancement in LF array is a superposition of various mechanisms; namely, light trapping associated with the array and possible scattering capabilities of a single LF^6^,^38^. The contribution of each mechanism is yet to be unveiled. Interestingly, the superior absorption of a single LF as compared with single NW can also be described when considering solely geometrical optics as was shown by Quinn^39^ who demonstrated the direct correlation between absorption and cone angle (α) with *A = 1 − T^n^* where *A* is the relative absorption, *T* is the relative reflectance of the cone wall and *n* is the number of the reflections a ray undergoes before it is reflected out of the cone. For incoming rays parallel to the axis of the cone *n = Int(π/2α)* where *Int(π/2α)* is the greatest integer less or equal to *(π/2α)*.

Figure 2c presents the simulated normalized absorbed photon density at wavelengths 600 nm, 720 nm and 840 nm along the vertical axis of a LF for a constant *D_t_* = 400 nm and *D_b_* = 100 nm and 200 nm and of a 400 nm NW for reference (all structures are nested in an infinite square-tiled arrays). Note that the NW exhibits low order modes, whereas the LF exhibit complex 3D modes. One can establish an intuitive understanding of this acute difference between the two types of structures by hypothesizing the swift transition from a NW geometry to the geometry of a LF; at a specific wavelength the hypothetical deformation of the NW into a LF modifies the constellation of internal reflections inside the cavity in such a way that renders the formation of complex 3D modes. Now, the presence of multitude complex 3D modes provides the strong coupling of light to the LF which is manifested in distinct absorption peaks.

Recently, two outstanding advancements in the field were published: the nanocylinder array^17^,^40^ and the nanocone array^16^ (reviewed above). We conduct comparative absorption simulations of the LF array against these two recent advancements in order to assess the contribution of the LF array to the state of the art. The dimensions and geometry of both the nanocylinder array and the nanocone array are taken directly from the publications (see methods). The simulated LF array is of the following geometry: square-tiled array with a 520 nm period, *D_b_* = 100 nm, *D_t_* = 400 nm, 2 μ height and 50 nm Si_3_N_4_ anti-reflection coating. Also, for reference a 2 μm thin silicon film with 80 nm standard anti-reflection coating is simulated. The aforementioned nanocylinder and the nanocones are relatively small features that require the presence of a substrate in order to maximize their performance and therefore the arrays are simulated on top of underlying substrates. The substrate thickness of the nanocylinder array is 2 μm to match the thickness of the 2 μm thin film. The substrates of the nanocone and the LF arrays are selected to ensure that all three technologies share the same amount of silicon, i.e. the height of the underlying substrates are 1.56 μm and 1.12 μm for the nanocone and the LF arrays, respectively. In order to crystalize the performance of the different arrays we applied perfectly matching layers boundary condition at the bottom of the substrates in order to exclude the contribution of the coupling of Fabry-Perot resonances with the waveguide resonances to the overall absorption. Henceforth, a reference to any array refers to the array together with the underlying substrate. Figure 2d compares the simulated relative absorption spectra of the LF array, the nanocylinder array, the nanocone array and the thin silicon film. First note that the thin film absorption is considerably higher than the absorption of the thin film in Fig. 2a due to the presence of the 80 nm Si_3_N_4_ anti-reflective coating. Also note the increase in absorption once the thin film is decorated with nanocylinder array (increase in ultimate absorption efficiency from 16% to 20%). The nanocone array exhibits a lower absorption as compared with nanocylinder array (ultimate absorption efficiency of 19%). However, the LF array presents a significantly superior broadband absorption with an ultimate absorption efficiency of 24.3% which reflects 52% improvement as compared with the thin film (this relative improvement differs from that in Fig. 2a due to the higher thin-film absorption on account of the 80 nm Si_3_N_4_ anti-reflective coating).

**Figure 3:**
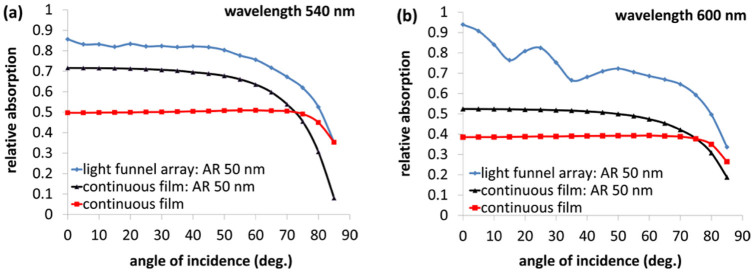
The dependency of the relative absorption on the angle of incidence for a continuous silicon film, a continuous silicon film with 50 nm Si_3_N_4_ antireflection coating and for a LF array with a period of 500 nm, *D_t_* = 400 nm an *D_b_* = 100 nm and with a 50 nm Si_3_N_4_ antireflection coating (all heights are 2 μm). These wavelengths are chosen as these are located at the vicinity of the peak power of the solar spectrum. (a), wavelength 540 nm. (b), wavelength 600 nm.

Photovoltaics require absorption enhancement over a wide range of angles of incidence (AOI). Figure 3a, b presents the AOI dependence of the relative absorption at wavelengths of 540 nm and 600 nm for a thin silicon film, a thin silicon film with a standard 50 nm Si_3_N_4_ anti-reflecting coating and for a LF array with *D_t_* = 400 nm, *D_b_* = 100 nm also with a standard 50 nm Si_3_N_4_ anti-reflecting coating (shown is the average of both polarizations). These two representative wavelengths are chosen as these are located close to the peak power of the solar spectrum. Note that the LF array presents an enhanced AOI dependency in both 540 nm and 600 nm wavelengths for the AOI range of 0–80 deg. The LF array maintains a relative absorption of ~85% whereas the coated thin film exhibits an absorption of ~70% for wavelength 540 nm in the AOI range of 0–50°. For AOI above 50° the absorption decreases for the LF array but is still substantially greater than the coated continuous silicon film.

### Electrical modeling

**Figure 4:**
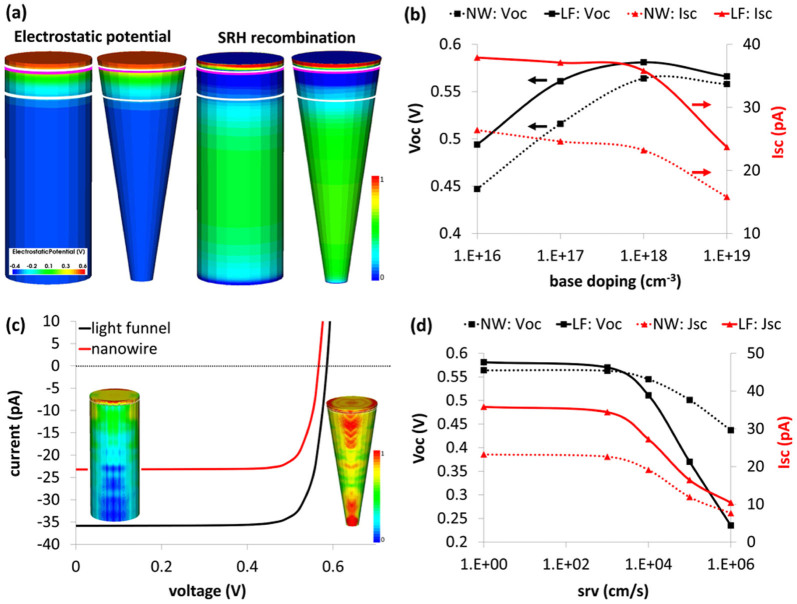
3D electrical simulations comparing the performance of a single LF with a single NW in their respective arrays under Air Mass 1.5 illumination. (a), Left. The electrostatic potentials inside a NW and a LF for 10^20^ cm^−3^ emitter (phosphorus) and 10^16^ cm^−3^ p-type base. Right. Normalized SRH recombination. (b), *V_oc_* and *I_sc_* vs. base doping for NW and LF solar cells. The main advantage of LF over NW is the increased *I_sc_* which is a consequence of the enhanced LF absorption. (c), Current vs. voltage of NW and LF for base doping of 10^18^ cm^−3^. The 3D NW and LF images presents the superior carrier generation in LF. (d), *V_oc_* and *I_sc_* of LF and NW vs. surface recombination velocity. Both structures are relatively insensitive to SRV in the range of 0–10^3^ cm/s. However for SRV > 10^3^ cm/s a sharper decrease in LF performance is recorded. This decrease is attributed to S/V effects.

3D TCAD simulations are also utilized in order to model the electrical response of the LF solar cell device. In the current investigation we also adapt the methodology employed in the optical section and perform an electrical comparison between the LF array and the NW array in order to better understand and describe the electrical behavior of the LF array. For this purpose the LF array of *D_t_* = 400, *D_b_* = 100 nm is selected and compared with a NW array with *D* = 400 nm. The calculations pertain to the opto-electronic performance of a single LF and a single NW nested in their respective arrays as the above optical modeling is performed for an infinite periodic square-tiled arrays. The main concern relating to the electrical performance of the LF is surface effects as the surface-to-volume ratio (S/V) of the LF is greater (by 75% for the selected LF and NW) and hence expected to be more sensitive to surface recombination velocity (SRV). In order to address specifically this matter we selected an axial junction configuration as it is more susceptible to surface effects than a radial structure^9^,^12^,^41^ (see methods). Figure 4a presents the solution of the Poisson equation (electrostatic potential distribution) and the distribution of Shockley-Read-Hall recombination (SRH) for both structures. The white and pink lines mark the depletion regions and the metallurgical junction, respectively, where the metallurgical junction is defined as the interface between the n and p region or, in other words, the interface where the concentration of the acceptors and donors is equal. Note the high recombination at the degenerated emitter and the low recombination at the depletion areas.

Figure 4b presents the open circuit voltage (*V_oc_*) and short circuit current (*I_sc_*) of a NW and a LF for a range of base doping levels and the respective SRH bulk lifetimes as the doping-dependent SRH recombination implies that bulk SRH minority carrier lifetimes (τ_SRH_) are coupled to the doping level^42^,^43^,^44^,^45^, and Fig. 4c shows the current vs. voltage (I–V) curves for base doping level of 10^18^ cm^−3^. For both devices *I_sc_* decreases with increase in base doping due to increase in SRH recombination (see Supplementary S3). Also in both cases *V_oc_* exhibits a maximum where it increases for base doping of 10^16^ cm^−3^–10^18^ cm^−3^ despite the decrease in *I_sc_*; the increase in base doping entails a decrease in saturation current and increase in *V_oc_* while the simultaneously decrease in *τ_SRH_* concludes an increase in saturation current and a decrease in *V_oc_*^46^. Note, that for base doping level of 10^19^ cm^−3^ the effect of recombination overcomes the gain due to an increase in doping and an overall decrease in both *I_sc_* and *V_oc_* is observed. The power *I_sc_* * *V_oc_* is maximized for base doping of 10^18^ cm^−3^ for both structures. At this base doping level the LF exhibits an increase of ~55% in *I_sc_* and ~3% in *V_oc_* relative to the NW device, where the relative superiority of the LF *I_sc_* over the NW stems from the superior light absorption. Finally, at base doping of 10^18^ cm^−3^ the LF presents an overall increase in *I_sc_* * *V_oc_* of 60% relative to the NW which reflects 60% increase in the PCE of the LF solar cell device over the NW-based device.

Figure 4d presents the dependencies of *V_oc_* and *I_sc_* for both devices on SRV (base doping of 10^18^ cm^−3^). *V_oc_* and *I_sc_* are insensitive to surface for both NW and LF in the range of SRV = 0–1 * 10^3^ cm/s. However, for SRV > 10^4^ cm/s a substantial decrease in LF *V_oc_* is shown. This is expected as the S/V ratio of the LF is higher than the S/V ratio of the nanowire by 75%. This is specifically evident when considering the model formulated by Allen et al^47^ for carrier lifetimes in NWs. Here, we easily translate this model to an oversimplified but insightful description of the LF where we slice the LF along the vertical axis and the following describes the relationship between the various lifetimes in each slice: 1/τ_eff_ = 1/τ_b_ + 4 * SRV/d_LF_(*z*) where *τ_eff_* is the effective carrier lifetime, *τ_b_* is the bulk lifetime and *d_LF_*(*z*) is the diameter of the LF at a certain location along its vertical z-axis. As *d_LF_* decreases toward the lower base of the LF so is *τ_eff_* which reflects the fact that at the bottom of the LF the surface plays a more pronounced role and hence the overall *τ_eff_* of the LF is smaller than that of the NW for SRV > 10^4^ cm/s. Specifically, *τ_eff_* of the NW and at the very top of the LF is 4 times greater than *τ_eff_* at the bottom of the LF (10^−9^ sec and 2.5 * 10^−10^ sec at the top and bottom of the LF, respectively). Finally, one should keep in mind that in order to avoid detrimental surface effects one could utilize radial junction configurations or apply appropriate surface passivation in order to suppress SRV as, for example, was already demonstrated with Al_2_O_3_ passivation where SRV < 10 cm/s was measured^48^.

As was performed in the above optical section, we compare the electrical performance of the LF array with recent advancements in the field, namely the nanocone and the nanocylinder arrays. Provided the LF array, the nanocone array and the nanocylinder array are transformed into solar cells with identical electrical configurations (i.e. same type of junction, same doping…) than all three cells will exhibit identical *V_oc_* and fill-factor. Therefore the increase in ultimate absorption efficiency is directly translated into an increase in PCE. This implies almost 30% PCE increase of the LF solar cell as compared with nanocone array that holds the record PCE for thin film solar cell. Remarkably, the LF array is superior to the nanocylinder array although the enhanced performance of the nanocylinder array as compared with a thin film is most significant when coupled to a thin substrate such as demonstrated here^49^. S/V ratio is another paramount parameter that needs consideration. For the optimized nanocone and nanocylinder geometries used in the optical section the S/V ratio of the nanocone and the LF are identical (15%) while the S/V ratio of the nanocylinder is higher (22%) (see methods), and therefore we expect the LF array to be less susceptible to SRV. Also, both the nanocone and the nanocylinder geometries cannot support the formation of a diffused homo-junction inside the nanostructures as these are too small, whereas the larger LF could also potentially accommodate this electrical configuration. Lastly, further improvements in the performance of the LF array could take place as the LF geometry used here is not fully optimized.

## Conclusions

We present here a new light trapping technique for photovoltaics based on LF arrays that mimics the structure and arrangement of the foveal cones. The absorption of the LF array is demonstrated for an array period of 0.5 μm where absorption enhancement of ~65% in comparison with a thin continuous silicon film of the same thickness is shown. The increase in absorption, as compared to a corresponding NW array, is attributed partly to superior light trapping coupled with an increase in the absorption efficiency factor of a single LF. Also, it is shown that the PCE of a single LF solar cell nested in an array is ~60% higher than the efficiency of the corresponding NW solar cell. The PCE increase is attributed to the increase in absorption with the consequential increase in *I_sc_* although the NW solar cell consists of more than 2.3 times the amount of silicon. We also demonstrate the superior absorption of the LF arrays as compared with recent advancements in the field. Finally, the fabrication of silicon LF arrays is exemplified using low-cost processing techniques.

## Methods

### Fabrication of light-funnel arrays

Silicon wafers were patterned using nano-sphere lithography. This process entails the formation of self-assembled monolayers of polystyrene nanopheres (PSS) on top of the wafers using the Langmuir-Blodgett technique. In the present case PSS of 1 μm in diameter were used. The resulting monolayers exhibit densely-packed hexagonal tiling that spans areas of several square centimeters. After deposition the size of the PSS was reduced with O_2_ plasma using Oxford Instruments Plasmalab 100 inductively coupled reactive ion etching. The silicon etch was performed with Oxford Instruments Plasmalab 100 inductively coupled plasma reactive ion etching system run in a cryogenic mode using a gas mixture of SF_6_, C_4_F_8_ and O_2_. Finally, the PSS were removed in an ultrasonic bath. See Supplementary S1 for more details.

### Numerical calculations

The optical and the electrical simulations were performed with Synopsys TCAD Sentaurus, Mountain View, CA, USA.

### Finite-difference time-domain (FDTD) electromagnetic simulations

The relative absorption, absorption efficiency factor and the field distributions were calculated using a three-dimensional finite-difference time-domain simulations. The simulation box size was set to the size of the unit cell with periodic boundary condition along the lateral dimensions and perfectly matching layers along the vertical axis (A perfectly matching layer is an artificial absorbing layer for wave equations that is used especially in FDTD to truncate computational regions in open boundaries problems). For each run the absorption, reflection and transmission were calculated using sensors that were located above the device (reflection) and below the device (transmission). In addition, for each simulation run the absorbed photon density and the power flux density were calculated at each mesh point. The calculations were performed for a plane wave excitation and a spectrum range of 400–1100 nm in 10 nm steps. The material optical constants were taken from the literature^50^. The details of the nanocone and the nanocylinder arrays were taken directly from the publications^16^,^17^. The nanocylinder array: square-tiled array with period of 450 nm, nanocylinder width of 250 nm, nanocylinder height of 150 nm, and 50 nm Si_3_N_4_ anti-reflection coating. The nanocone array was simulated with the following: square-tiled array with a period of 500 nm, nanocone bottom diameter of 450 nm, nanocone height of 400 nm and 80 nm coating of SiO_2_ (note that Jeong et al measured hexagonal array^16^).

### Electrical simulations

The Poisson and the Continuity equations were solved for each mesh vertex in conjunction with the carrier generation file that was calculated from the absorbed photon density. Top and bottom contacts were defined and forced the respective biasing as boundary conditions. The simulated axial devices are composed of a top degenerately phosphorus-doped emitter (n-type) and a boron-doped base (p-type) (Fig. 4a). The emitter is 200 nm–300 nm in height with an error function dopant distribution to account for a standard implant process^51^. The degenerated emitter prohibits the generation of photocurrent due to the high levels of recombination and hence serves solely for the formation of a pn-junction and as a top ohmic contact and therefore confined to a small volume as realistic implant/diffusion processes render possible. The absorbed photon density reflects the weighted absorption once the devices are subjected to solar spectral irradiance of Air Mass 1.5 (as performed in the optical part). The optical generation is calculated in the following manner: for excitation energy greater than or equal to the bandgap energy, the quantum efficiency is set to one and otherwise, it is set to zero. For each simulation mesh point the Poisson and the Continuity equations are solved where doping dependent Shockley-Read-Hall (SRH) recombination, Auger recombination, surface recombination, bandgap renormalization for degenerately doped silicon and doping dependent mobility, are all accounted for. It is assumed that the starting material is standard Czochralski silicon which is reflected in the selection of the following parameters. The rate of SRH is calculated in the following manner^46^:
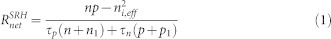
where *n* and *p* are the respective electron and hole carrier densities, *n_i,eff_* is the effective intrinsic density (accounting for bandgap narrowing), *τ_n_* and *τ_p_* are the respective electron and hole lifetimes and *n_1_* and *p_1_* are:
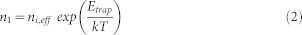

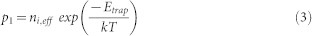
where *E_trap_* is the difference between the defect level and the intrinsic level and is set to zero in the current case. The dependency of SRH on doping is reflected in the dependency of carrier lifetime on doping:
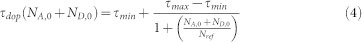
where *N_A,0_* and *N_D,0_* are the chemically active acceptor and donor concentrations, respectively. In the present case *γ = 1*, *τ_min_* = 0, *N_ref_* = 10^16^ cm^−3^ for both holes and electrons and *τ_min_* = 10^−5^ sec and 3 × 10^−6^ for electrons and holes, respectively.

The rate of Auger recombination is calculated in the following manner^46^:
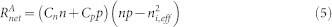
where *C_n_* and *C_p_* are set to 2.9 × 10^−31^ cm^6^s^−1^ and 1 × 10^−31^ cm^6^s^−1^, respectively^52^.

## Supplementary Material

Supplementary Information Supplementary information
