# Supplementary material for: Enhanced photovoltaics inspired by the fovea centralis
Source: Sci Rep. 2015 Feb 24;5:8570. doi: 10.1038/srep08570 (PMC4338419; doi:10.1038/srep08570)
Supplement: Supplementary Information [file srep08570-s1.docx]

**Enhanced photovoltaics inspired by the fovea centralis**

Gil Shalev, Sebastian Schmitt, Gerald Brönstrup, Heidemarie Embrechts, Silke Christiansen

**Supplementary S1**


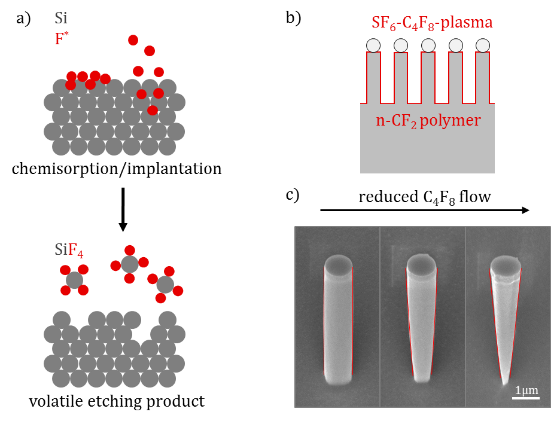


A stable and directional selective cryogenic RIE process based on the process gases SF_6_, O_2_ and C_4_F_8_ was developed for the fabrication of vertically aligned SiNW arrays from Si samples masked with polystyrene nanospheres ^1^. To permit a high stability of the etching reaction, the sample is cooled to liquid nitrogen (LN_2_) temperature. In the ICP-RIE process, the basic reaction for the Si decomposition is given by


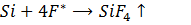


where Si and fluorine ions F* react to volatile silicon tetra fluoride (SiF_4_). The details of the reaction process are shown in figure (a). Fluorine ions created in the ICP can either be brought to reaction with Si through chemisorption at the surface, or through implantation into the Si crystal. While surface-near Si ablation by the chemisorption reaction is slow and isotropically distributed over the surface, Si ablation through fluorine ion implantation is directional and comparatively fast, since fluorine ions are implanted into deeper planes of the Si lattice and additionally cause mechanical damage (physical etching). The difference in etching speed for the two described Si ablation mechanisms therefore induces an inherent directional selectivity to ICP-RIE processes that allows to etch high aspect ratio structures such as SiNWs by lithographic masking of the sample surface. The selectivity is further enhanced by a sidewall passivation of the structures that reduces ablation of Si by the isotropic chemisorption of fluorine ions. In an SF_6_-C_4_F_8_ plasma chemistry this passivation is provided by the surface formation of an n-CF_2_ polymer (figure b). In a receipt for the etching of straight SiNW which is specific for the used reactor and the processed sample, the sidewall taper (degree of under etching) of the SiNW can now be tuned by a reduction of the gas responsible for passivation. Figure (c) shows the increased sidewall taper of SiNW caused by a C_4_F_8_ flux reduction in an SF_6_-C_4_F_8_ plasma. Remaining silica spheres used for lithography and surface roughness of the as etched samples are removed by an HF dip after the etching process.

**Supplementary S2**

The figure below presents the *Q_abs_* of three NWs: D=400 nm and period of 2 µm, D=400 nm and period=0.5 µm and D=100 nm and period=0.5 µm (all with height of 2 µm). The NW D=400 nm and period of 2 µm reflects a relatively isolated NW and hence the higher *Q_abs_* across the full spectra as compared with the NW of D=400 nm and 0.5 µm which reflects a *Q_abs_* of a NW nested in an array. Therefore, once a NW is relocated from an isolated environment into an array its *Q_abs_* drops significantly; in the current study case the average *Q_abs_* drops from 0.9 for the isolated NW to *Q_abs_*=0.63 for the nested NW. The loss of *Q_abs_* can be recovered by reducing the size of the diameter, as shown in the figure, where the NW of D=100 nm and period=0.5 µm has an average *Q_abs_* of 3.9. (However, one should keep in mind that the overall absorption of the 100 nm NW array will be smaller than the array absorption of the D=400 NWs.).


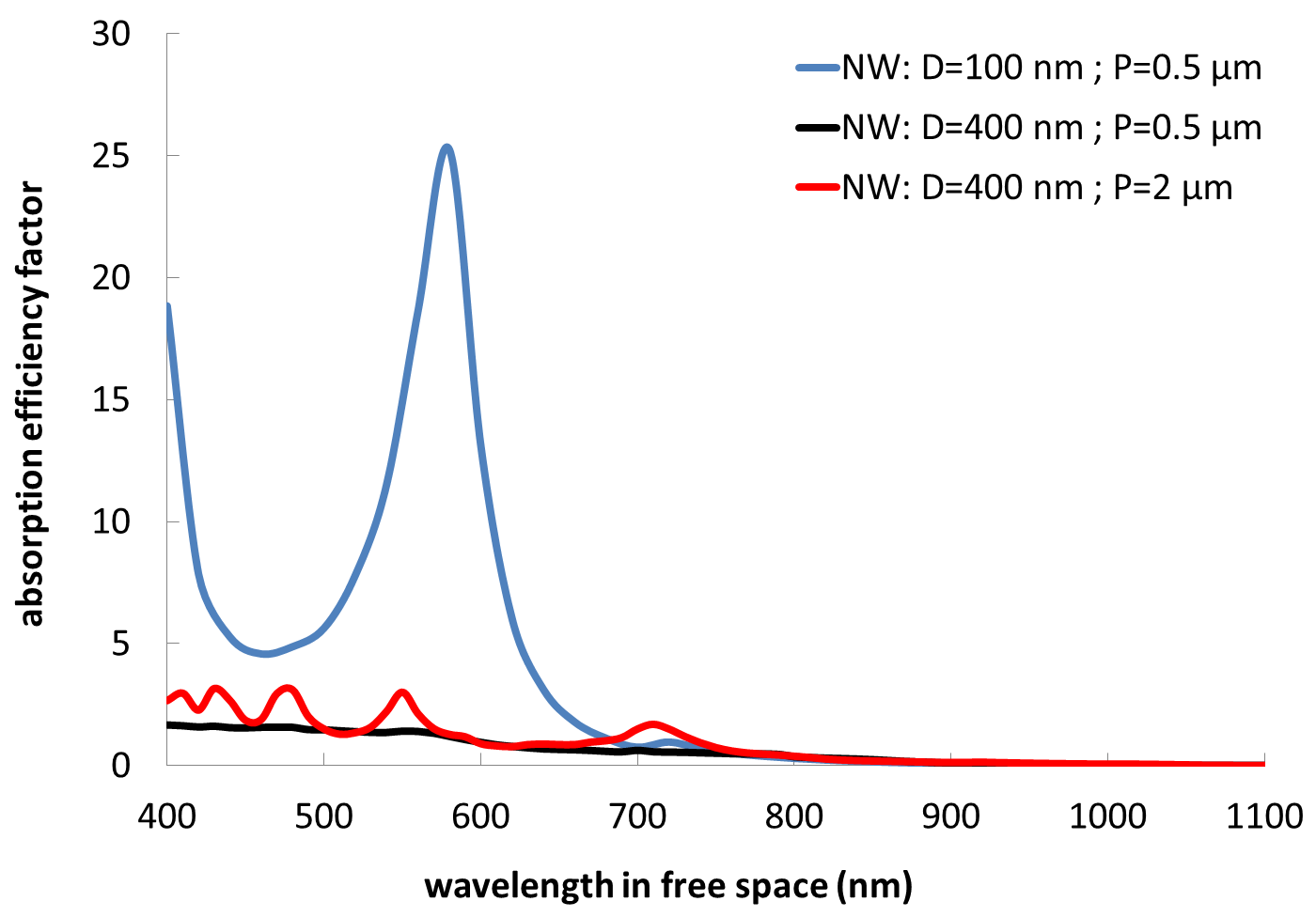


**Supplementary S3**

SRH recombination and Auger recombination as a function of base doping level for both LF and NW.


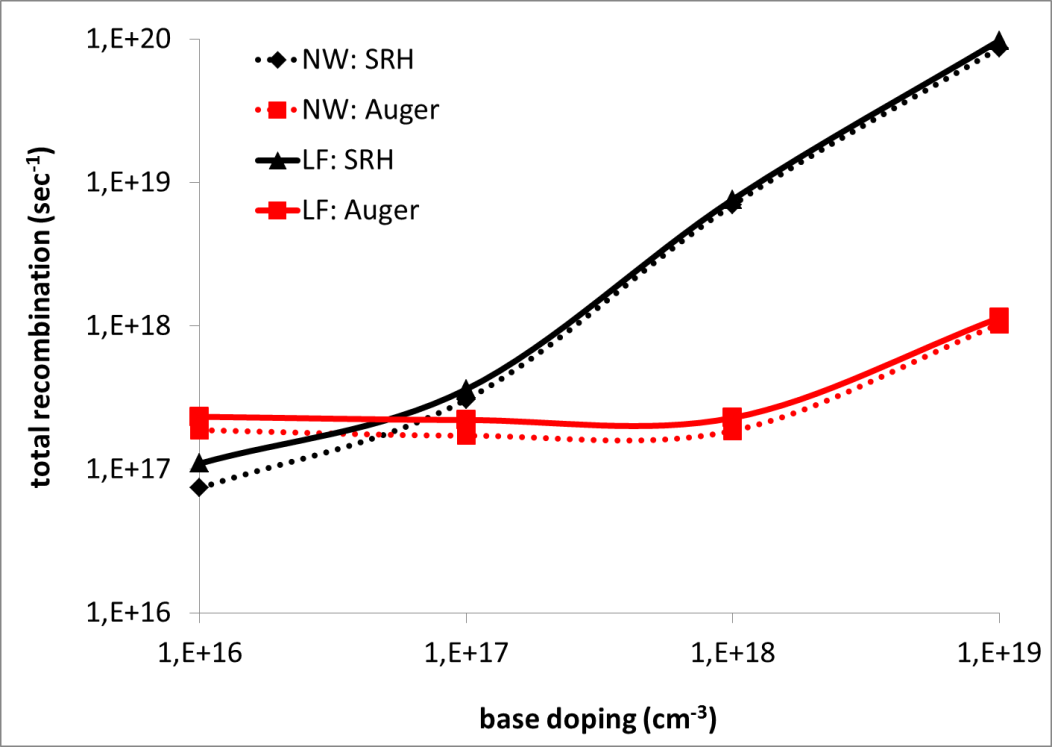


**References**

1. Schmitt, S. W. *et al.* Nanowire arrays in multicrystalline silicon thin films on glass: a promising material for research and applications in nanotechnology. *Nano Lett.* **12,** 4050–4054 (2012).
